# Manual of British Botany

**Published:** 1838-10

**Authors:** 


Art. II.—*Manual of British Botany.* By D. C. Macreight, m.d., Fellow of the Royal College of Physicians, &c.—*London,* 1837. 12mo. p. 296.

This little work, arranged upon the natural system of De Candolle, attracted our attention as promising to supply a want increasingly felt amongst botanical students. The synopsis of Dr. Lindley, although in general strictly scientific, and devoid of obscurities of any moment to the initiated reader, requires, nevertheless, such a precision in the application of the concise analysis of orders as to frustrate, not seldom, the sedulous investigation of the student. But in the manual before us, the compiler does not appear to have lost sight of the fact, that few of his readers in this country will be accomplished adepts in the natural system: indeed his generous accommodation of himself, as the advocate of the school of De Candolle, to the dawning intelligencies of those who are just emerging out of the nursery of Linnæus, is strikingly manifested by the desire which he expresses, (*Preface*, p. 2,) that those who cannot read and distinctly pronounce his brief definitions of species, should at once go back and spell out their lessons in the dame-school of Dr. Hooker. We doubt not that in Dr. Lindley's view, such amiable liberality to the Linnæans must savour of absolute heresy; and in truth, if Dr. L. be just in his opinion (see preface to Synopsis,) that the language of this school is far from accurate: "that terms are applied in their works so vaguely and erroneously, and that they so abound in mistakes, most of which are at variance with all correct notions of the structure of plants, that they are totally unfit to be placed in the hands of students;" Dr. Macreight will have disserved those whose advantage he expressly contemplates, in referring them to the British Flora of Dr. Hooker for more diffuse information upon species.

We have examined the analytical tables of the manual in two or three of the more intricate and important orders, and we should conceive that diligent attention may lead the student in general safely to the species required, and to a correct view of both the order and genus to which it belongs. Dr. Lindley's Synopsis possesses, however, one advantage in a concise *specific character* attached to the name of each plant, which must still recommend his work to those who have not leisure or inclination for a prolix analytical examination of species. We heartily desire that Dr. Macreight's little work may find acceptance with those who admire elaborate ingenuity and faithfulness to principles. The author will have reason to note several *corrigenda* in a second edition.

